# Recent advances in understanding the roles of whole genome duplications in evolution

**DOI:** 10.12688/f1000research.11792.2

**Published:** 2018-03-29

**Authors:** Carol MacKintosh, David E.K. Ferrier

**Affiliations:** 1 Division of Cell and Developmental Biology, University of Dundee, Dundee, Scotland, DD1 5EH, UK; 2 The Scottish Oceans Institute, University of St Andrews, Scotland, KY16 8LB, UK

**Keywords:** WGD, Whole-genome duplications, evolution, Ecology, Biodiversity, Polyploidy

## Abstract

Ancient whole-genome duplications (WGDs)— *paleo*polyploidy events—are key to solving Darwin’s ‘abominable mystery’ of how flowering plants evolved and radiated into a rich variety of species. The vertebrates also emerged from their invertebrate ancestors via two WGDs, and genomes of diverse gymnosperm trees, unicellular eukaryotes, invertebrates, fishes, amphibians and even a rodent carry evidence of lineage-specific WGDs. Modern polyploidy is common in eukaryotes, and it can be induced, enabling mechanisms and short-term cost-benefit assessments of polyploidy to be studied experimentally. However, the ancient WGDs can be reconstructed only by comparative genomics: these studies are difficult because the DNA duplicates have been through tens or hundreds of millions of years of gene losses, mutations, and chromosomal rearrangements that culminate in resolution of the polyploid genomes back into diploid ones (rediploidisation). Intriguing asymmetries in patterns of post-WGD gene loss and retention between duplicated sets of chromosomes have been discovered recently, and elaborations of **signal transduction **systems are lasting legacies from several WGDs. The data imply that simpler signalling pathways in the pre-WGD ancestors were converted via WGDs into multi-stranded parallelised networks. Genetic and biochemical studies in plants, yeasts and vertebrates suggest a paradigm in which different combinations of sister paralogues in the post-WGD regulatory networks are co-regulated under different conditions. In principle, such networks can respond to a wide array of environmental, sensory and hormonal stimuli and integrate them to generate phenotypic variety in cell types and behaviours. Patterns are also being discerned in how the post-WGD signalling networks are reconfigured in human cancers and neurological conditions. It is fascinating to unpick how ancient genomic events impact on complexity, variety and disease in modern life.

## Comparative plant genomics help solve Darwin’s abominable mystery

Darwin was vexed. While natural selection could explain gradual evolutionary transitions, the apparent sudden appearance of diverse flowering plants in the Cretaceous fossil record of approximately 130 million years ago (Mya) was his ‘abominable mystery’ ^1^. Fast-forward to today’s exciting era of high-throughput genome sequencing, and phylogenomic maps assembled from multiple whole-genome sequences tell the evolutionary story with revised timelines:

From the Carboniferous to early Cretaceous periods (approximately 360 to 130 Mya), the land was dominated by gymnosperms (literally ‘naked seeds’) including the cycads, Ginkgo, and conifers that still flourish in subarctic forests ^2^. The nuclear genomes of several gymnosperms have been sequenced recently, a heroic undertaking, given their exceptional size (10 to 40 gigabases) and high density of long terminal repeat (LTR)-retrotransposon repeats ^3– 5^. Within these genomes many non-overlapping duplicated chromosomal regions were identified that display gene synteny, meaning that their gene contents are similar to those of other chromosomal blocks within the same genome and across gymnosperm genomes. These gene synteny patterns and complementary transcriptome data support the hypothesis that the gymnosperms emerged from their common ancestor via a WGD named ζ that occurred an estimated 390 Mya during the Devonian period ^6– 9^. Tell-tale traces of further lineage-specific WGDs have also been discovered in the genomes of Norway spruce, Sequoia and Ginkgo, and in the unusual two-leaved Namibian *Welwitschia,* suggesting that multiple WGDs contributed to the diversity of these gymnosperms, which include the longest-living and largest organisms on Earth ^3– 5, 7^.

Flowering plants (angiosperms, ‘seed born in a vessel’) are the most abundant plant group today, having a rich diversity of some 400,000 species from bananas to water lilies, grasses to beech trees ^10^. Many angiosperm genomes have been selected for sequencing to discover genes for special agronomic traits. The resulting genome assemblies have been used collectively to extrapolate back in time, tracking the genome evolution of the monocot and eudicot angiosperms, as well as more primitive flowering plants, and converging on a reconstructed genome of the most recent common ancestor of all angiosperms ^6^. These new phylogenomic maps solve part of Darwin’s dilemma by confirming that the first flowering plants evolved between 140 and 250 Mya after an unknown gymnosperm went through a WGD (named the ε event) an estimated 300 Mya during the Carboniferous period ^6, 8, 9^. The clearest support for the ε WGD comes from multiple gene synteny blocks in the genome of the primitive angiosperm *Amborella trichopoda*, whose ancestral lineage diverged early on from other flowering plants, experiencing no further post-ε WGDs ^11^.

The antiquity of the ε WGD means that angiosperms had the entire Triassic and Jurassic periods to evolve and diversify into the species richness reflected in the fossils of approximately 130 Mya that were known to Darwin. Aligning with the recalibrated timelines, older angiosperm microfossils have been discovered, though claims that the earliest ones date to the Triassic are controversial ^12, 13^. In any case, angiosperm evolution was not as sudden an explosion as Darwin thought, and now a comprehensive phylogenomic framework exists to mine for answers about how angiosperm complexity and variety evolved after the ε WGD.

## Contributions of whole-genome duplications to the origin and diversity of flowers

In the 1970s, Susumo Ohno had the prescience to propose that evolutionary leaps could occur by WGDs because one of each gene pair may continue to do what it was doing before, giving freedom for the other to either be lost from the genome or to evolve new characteristics (neofunctionalise) ^14^. Members of gene families generated via WGDs are named ohnologues in his honour ^15^. Another scenario is for the duplicates to each retain subsets of the functions of the ancestral gene (subfunctionalisation) ^16^. Moreover, the *en masse* diversification of many gene duplicates after a WGD would be expected to create selective pressures between gene families and opportunities for new interactions among them, so that large duplicated gene and protein sets co-evolve as complex systems.

Floral organs provide a canonical example of how interacting sets of diversified ohnologues can make variant structures. While many gene pairs lost one duplicate after the ε WGD, the retained ohnologue pairs include MADS-box transcription factors. Some of these were characterised more than 20 years ago for their ability to interact with each other in different combinations to specify the floral organs of *Antirrhinum* (snapdragon) and *Arabidopsis*. In these seminal studies, homeotic floral mutants were used to deduce an elegant ‘ABC model’, later elaborated into an ‘ABCDE model’ that explains how the four concentric whorls of sepals, petals, male stamens and female carpels develop in these flowers ^17– 19^. A-function genes specify sepals; A, B and E are needed to make petals; B, C and E male stamens; C female carpels; and D for ovules. In homeotic mutants lacking B-function, for example, sepals replace petals and carpels replace stamens ^18^. Most of the A, B, C, D and E functions are performed by combinations of MADS-box transcription factors that operate as homodimers and heterodimers and tetramers with different selectivities for binding to variants of a common motif in the promoters of target genes ^19, 20^. Hence, they target overlapping but distinct sets of floral identity genes many of which are themselves ohnologues ^21^.

The recent genome comparisons indicate that male ‘BC’ and female ‘C’ systems already existed to specify reproductive cells in gymnosperm cones, and they were duplicated via the ε WGD, after which the C duplicates diversified into angiosperm C and D genes. An A/E gymnosperm pro-orthologue gave rise to angiosperm A and E genes, and further duplicated A genes were also retained after the ε WGD ^19, 21– 24^. These duplicated and diversified gene sets organised to generate the first now-extinct flowers, and recent reconstructions suggest that these were bisexual with petal-like tepals and pollen-bearing stamens arranged in multiple concentric whorls, and female carpels in a central spiral ^25^. Among living angiosperms, the *Amborella* lineage evolved a ‘fading borders’ programme, such that the whole flower is a spiral that gradually transitions from bracts to outer then inner tepals (specified by ABc combinations), from inner tepals to stamens (aBC) then carpels (abC), in which upper case indicates functions of greatest influence in the respective organs ^26^. Only in later-evolving flowers such as *Arabidopsis* and *Antirrhinum* did the tepals subdivide into sepals and petals, by restricting the boundaries of expression of floral identity genes. For example, the transcription of A and C genes became mutually exclusive ^26^. Further evolutionary diversity in flower form occurred by mechanisms that include shifts in the spatial expression of ABC functions across flowers, and by further WGDs that elaborated and extended the ABC regulatory network ^27^. For example, in stylised orchid flowers, subfunctionalisation of duplicated B genes underpins the development of three types of petals: three outer tepals, two inner tepals and a modified lip ^28^. Recent case studies implicate additional ancient WGDs – including one at the base of the eudicots, the γ genome triplication in the Pentapetalae (five-parted, the largest flower clade), and ρ, σ and τ polyploidisations for monocots – in evolution of the phenomenal variety of architectural form and size in pollen, fruits and seeds, in diversification of plant defence metabolites, and in the co-evolution of angiosperms with pollinators and symbiotic bacteria ^6, 29– 34^.

## Mechanisms and cost-benefit analyses in recent natural and experimental polyploidies

Evolutionarily recent polyploidy events are also prevalent in flowering plants. Many crops including coffee, bananas, peanuts, tobacco, kiwifruit and strawberries were unwittingly selected as polyploids for their exaggerated traits such as large fruits, seeds and leaves ^35^. For example, the durum wheat used to make pasta is a tetraploid resulting from hybrid doubling of the genomes of two diploid wild-grass ancestors approximately 0.5 Mya, and was selected for domestication much later by Neolithic farmers, during which time hexaploid bread wheat emerged by hybridisation of the tetraploid with a diploid followed by another WGD ^35^. Like wheat, many well-characterised crop polyploids are allopolyploid ^36^, which means that the genome became polyploid after a hybrid was formed between species, in which case the WGD resolved problems with meiotic pairing by providing each chromosome with a homeologous partner ^37, 38^. However, autopolyploidy events, self-duplication within a species, are suspected in the ancestry of potatoes, bananas, poplar and soybean ^39^.

Recent statistical comparisons suggest that an individual polyploid plant has a higher risk of extinction than its still-diploid relatives ^40– 43^. It makes sense that the odds are stacked against a newly tetraploid plant. Breeding with still-diploid relatives results in triploid progeny that cannot separate evenly into two gametes during meiosis, most often resulting in sterile offspring, and unless self-pollination can occur, chances may be low of finding compatible polyploid mates. Farmers get around such problems by cloning: grafting apple trees or propagating potato tubers instead of seeds, whilst sterility (for example in seedless bananas) is sometimes even preferred ^44^. However, the resulting monocultures may be susceptible to pathogens, as when the *Fusarium oxysporum* fungal pandemic brought the popular triploid Gros Michel banana to the brink of extinction in the 1960s ^45^.

Nevertheless, their prevalence suggests that once polyploids have beaten the early survival odds, with or without human intervention, their polyploid traits such as larger organs, stress tolerance and altered flowering time may improve fitness or allow them to adapt to new ecological niches ^46^. Experimental polyploidies show that having extra DNA can produce an immediate phenotypic change, attributable to gene dosage effects. For example, dwarfism in apple plants with colchicine-induced autotetraploidy correlates with increased expression of a microRNA that acts via a gene regulatory network to downregulate synthesis of auxin and brassinosteroid growth regulators ^47^. More generally, polyploid plants are notable for their increased cell and organ size, which is more than a passive consequence of increased nuclear DNA content: in experiments with *Arabidopsis,* increases in cell volume upon tetraploidisation were found to vary in different mutants and according to cell type, indicating a genetic contribution ^48^.

For allopolyploids, the relative contributions of the species hybridisations and the WGDs to subsequent evolution are interwoven. In a recent molecular dissection of the circadian clock in allotetraploids formed between diploids *Arabidopsis thaliana* (At) and *Arabidopsis arenosa* (Aa), biases in heterologous combinations of components were discovered: the Aa-derived CCA1 hiking expedition (CHE) transcription factor preferentially binds to the promoter of the At circadian clock associated 1 ( *CCA1*) gene, elevating its expression over that of the AaCCA1. Such biased patterns of expression, and of protein–protein and protein–DNA interactions in the circadian regulatory network make the rhythm of the allotetraploid distinct from that of either parent ^49^.

## Common themes from recent reconstructions of ancient whole-genome duplications in plants, animals and fungi

Successful polyploidy is said to be less common in animals than in plants. Based on incidences of chromosomal anomalies in embryos that fail to develop, it appears that when two sperm fertilise one egg or when meiotic cell division fails the result is usually lethal in humans and birds ^50, 51^. However, polyploidy is relatively common in ectothermic vertebrates. Also, synthetic fish and shellfish polyploids, generally sterile, have been created to increase food production ^52^. Moreover, helped by technical advances in deep sequencing, genome assembly and pattern-matching software, ancient WGDs have been identified in invertebrate and vertebrate animal lineages, including mammals. The most recent discovery was that the house spider *Parasteatoda tepidariorum* and bark scorpion *Centruroides sculpturatus* are common descendants of a WGD that occurred over 450 Mya, and was distinct from an ancestral WGD of horseshoe crabs ^53, 54^.

Although few ancient WGDs have been identified thus far in unicellular eukaryotes, the diploid baker’s yeast *Saccharomyces cerevisiae* and five other fungal genera all stem from the same well-characterised ancestral allopolyploid WGD approximately 100 Mya ^55^, and further fungal WGDs have been identified recently. For example, the opportunistic honeybee fungal pathogen *Nosema ceranae*, which is spreading to beehives worldwide, is a suspected tetraploid ^56^.

In summary, ancient WGDs that were successful in leaving modern descendants have occurred in diverse eukaryotes across eons of time, in terrestrial and aquatic environments. Remarkably, despite their radically different contexts and a sparsity of data on ancient WGDs outside of laboratory models and domesticated species ^57^, common principles are emerging that tie disparate WGD events together:

- *Successive WGDs have occurred in multiple lineages:* As indicated, angiosperms have experienced multiple WGDs, and recursive WGDs among the *Brassica* crops that include cauliflower, broccoli and cabbages have been precisely mapped recently ^58^. The vertebrate animals emerged from the invertebrates approximately 500 Mya via two sequential rounds of WGD (2R-WGD) ^59^. In fish there was a further teleost-specific WGD (TSGD, 3R) approximately 300 Mya, followed by a salmonid-specific WGD (Ss4R) approximately 95 Mya; and certain *Xenopus* frog species and the red viscacha rat *Tympanoctomys barrerae* also result from lineage-specific WGDs ^60– 64^. The unicellular ciliate *Paramecium tetraurelia* has a history of three successive WGDs ^65^, and WGDs may have contributed to the record number of chromosomes (2 *n*=1260) in the fern-like genus *Ophioglossum* ^66^.
- *Most, though not all, well-characterised WGDs were allopolyploidy events:* In common with most polyploid plants, the tetraploid frog *Xenopus laevis* and diploid yeast *S. cerevisiae* were recently identified to be descendants of allopolyploidies ^67, 68^. In contrast, high similarity between homeologous regions in salmonid genomes indicate that the TSGD/3R and salmonid Ss4R were autopolyploidy events ^69, 70^. The mechanisms of the 2R-WGD at the origin of the vertebrates 500 Mya are unresolved, although early studies argued for two closely spaced autotetraploidies ^71^.
- *Long lag periods may occur between WGDs and subsequent species radiations*: A WGD generates a new organism that is immediately distinct from the parental species, especially after inter-species hybridisation allopolyploidies. Intuitively, one would expect this new organism to lead to species radiations due to the availability of new genetic material for evolution to mould in different ways. In practice however, the mechanistic links between WGDs and species radiations are not so clear-cut. In many instances there is a time-lag between WGDs and species radiations, formalised as the WGD Radiation Lag-Time model ^72^. For example, comparisons of post-TSGD fishes, including zebrafish and Japanese medaka, show that post-TSGD genome changes were biphasic. An initial period of bulk losses of chromosomal segments was overlaid by a more extended period of gradual gene losses by pseudogenisation and mutation of the retained ohnologues. The latter phase, after the initial bulk genome reshaping, correlates with radiation of bony fish species ^62^, though how or whether WGD is mechanistically linked to fish diversification is still an open question ^61, 73^. Among the fungi, comparisons of the six genera that share the same ancestral WGD as *S. cerevisiae* suggest that around 4000 genes still existed in duplicate when these genera were diverging from each other, with subsequent losses of different paralogues in different lineages, such that, for example, *S. cerevisiae* now has 551 pairs of ohnologues and *Candida glabrata* has 404 pairs ^55, 67, 74^. The next two bulleted points further discuss how mechanisms of post-WGD genomic evolution, as well as environmental influences, steer the course of speciation and phenotypic diversity after a WGD.
- *Post-WGD chromosomal rearrangements culminate in a return to the diploid state (rediploidisation) as well as lineage divergence:* Allopolyploidy results in immediate rediploidisation (diploid pairing of homeologous chromosomes during meiosis/mitosis) if the chromosomes from the two parental species are sufficiently distinct that chromosomes do not form quadrivalents during cell division. In contrast, autopolyploidy leads into a process of gradual rediploidisation ^37^, such that descendants of some WGDs that happened just tens of Mya are still polyploid, whereas species whose last WGD occurred hundreds of Mya (certain angiosperms and most vertebrates) have reverted to diploid. The post-Ss4R salmonids provide interesting snapshots of genomes in transition – some chromosomes are still functionally tetraploid whereas others have become diploid. Examining these genomes in different species reveals that the dynamic interplay of rediploidisation, ohnologue divergence, speciation and post-speciation evolution is complicated ^75^. For example, regions of salmonid genomes have been identified for which rediploidisation and evolutionary diversification of ohnologues occurs after speciation, such that functional divergence of ohnologues occurs in lineage-specific ways (Lineage-specific Ohnologue Resolution, LORe). LORe may facilitate adaptations of the distinct species to different ecological contexts ^76^.
- *WGDs and species radiations following WGDs have been linked with major climate change:* Many angiosperm WGDs date to the asteroid-triggered Cretaceous-Paleogene boundary events approximately 66 Mya, indicating that polyploid establishment may be favoured during times of environmental stress ^77^. After the Ss4R of approximately 88 Mya, the greatest species radiation occurred in fish that evolved the ability to migrate between fresh water and seawater following the climatic cooling of the Eocene–Oligocene transition 40 to 50 million years later ^78^. Such correlations between polyploidisations and environmental changes strengthen the view that the two are linked, perhaps due to polyploid organisms being more robust to environmental change and stress ^46^.
- *Knowledge of WGDs informs how laboratory animals are used as biomedical models*: Zebrafish and polyploid *Xenopus* frogs are valuable models for development and disease. However, they have been through lineage-specific WGDs that humans have not, which means that phenotypes may differ when ohnologues are mutated in zebrafish, polyploid frogs, and humans ^60, 79^. Moreover, families of sister ohnologues from the 2R-WGD may subfunctionalise or neofunctionalise in different ways along different vertebrate lineages, as has been found for the neurogenin and snail/slug genes ^80, 81^. It is therefore important to consider the composite functions of all sister ohnologues when making cross-species comparisons.
- *Asymmetries in the fates of DNA duplicates occur at multiple levels after ancient allopolyploidies:* Patterns of gene loss, retention and mutation may differ markedly in the sub-genomes derived from each parent of the original hybrid ^82^. A striking example is the tetraploid *X. laevis* in which large- and small-scale losses of DNA differ to such an extent that chromosomes derived from one parental species are markedly shorter than chromosomes from the other ^60^. It has been proposed that biases in the fates of DNA duplicates may result from ancient allopolyploidies, but that more evenly balanced post-WGD patterns of gene loss, retention and differentiation may follow on from ancient autopolyploidies ^39^. The proposal is that initial differences in expression levels and in affinities of interactions of ohnologue proteins derived from two parental species ^49^ could propagate into further knock-on biases such as preferential retention of highly-expressed genes ^65, 66^. Another type of post-WGD bias occurred in the shared ancestry of *S. cerevisiae* and *Candida glabrata.* Their ancestral WGD occurred approximately 100 Mya, after one parent from the KLE ( *Kluyveromyces*, *Lachancea*, *Eremothecium*) clade mated with one of the ZT ( *Zygosaccharomyces*, *Torulaspora*) clade. However, for various reasons the mixed parentage of *S. cerevisiae* was not immediately obvious when its genome was sequenced. One reason is that the *S. cerevisiae* genome contains more ZT-than KLE-derived sequences, possibly due to biased gene conversion that replaced some KLE-derived sequences with homeologous ZT-derived ones ^67, 68^. Finding that *S. cerevisiae* results from an allopolyploidy, rather than an autopolyploidy as originally believed, means that calculations of the relative rates of evolution of its ohnologue pairs ^82^ may need revision.
- *Retained ohnologue gene families are strikingly enriched in signalling and regulatory proteins in plants, fungi and animals* ^8, 82– 85^: For example, ancestral WGDs that were identified recently for the fungi *Mucor circinelloides* and *Phycomyces blakesleeanus* resulted in increased proportions of genes whose transcription is regulated by light ^86^. In humans, while only approximately 25% of genes are ohnologues stemming from the 2R-WGD, ~66% of protein kinases and nearly 90% of well-characterised 14-3-3-binding phosphoproteins are ohnologues; and developmental regulators and post-synaptic density (PSD) brain proteins are also highly enriched in ohnologues ^87– 92^. Signalling in biology ranges from simple signal-response systems to the complex signalling networks of our brains that coordinate complicated actions, create memories and find meaning in patterns. How have WGDs shaped these networks?

## Post-whole-genome duplication evolution of parallel processing via duplicated signalling networks, and dysregulation in cancers and neurological disorders

Studies in *S. cerevisiae*, plants and mammals have shown that regulatory proteins that form oligomers, that interact transiently with multiprotein complexes, and catalyse consecutive steps in metabolic and regulatory pathways are enriched among duplicate pairs that are retained following a WGD ^93, 94^. These findings are interpreted by the gene balance hypothesis, which states that copy numbers of genes encoding multi-protein structures and pathways must be kept in a constant ratio to avoid architectural disruption or metabolic imbalances ^95^, although stoichiometry can also be achieved by other mechanisms such as differential degradation of protein components ^95^.

Interestingly, the architectures of sister ohnologue proteins are generally conserved with respect to content and order of their domains, at least in vertebrates. Instead, functional divergence between sisters occurs via small-scale mutations that lead to differences in temporal and spatial patterns of expression, altered sites of regulatory post-translational modifications, and changes in specificities and affinities of catalytic domains and interaction interfaces ^88, 92, 96, 97^.

Examples abound to illustrate how the resulting families of differentiated sister ohnologues contribute to phenotypic robustness, plasticity and complexity. For instance:

- The ‘Gli code’ refers to how different combinations of sister Gli transcription factors—Gli1 and Gli2 that activate transcription and the repressor Gli3—influence tissue shape and size during vertebrate embryonic development. The code can be changed via differential regulation of the sister Gli proteins by multiple signalling pathways ^98^.
- Genetic and biochemical dissections indicate that sets of sister ohnologues within the PSD ‘supercomplexes’ of mammalian brains can differentially process the signals responsible for individual cognitive abilities and emotions ^99^. For example, in mammals the discs large MAGUK scaffold protein 4 (Dlg4) has evolved a role in simple associative learning, whereas its sisters Dlg2 and Dlg3 have distinct and opposing functions in complex cognitive processes ^87^.
- In *S. cerevisiae,* differential pathways can be created by co-ordinated expression of different combinations of ohnologues that have been largely partitioned into obligate sub-networks ^100^. Switching from one sub-network to another allows the yeast to adapt to different stresses, and to reconfigure fluxes through metabolism to enable growth on different types and quantities of sugars ^100– 104^.
- The phosphoprotein-binding 14-3-3 proteins interact with hundreds of (phosphorylated) ohnologue proteins in mammalian cells, suggesting that regulated phosphorylation of 14-3-3 docking sites provides a large-scale mechanism for switching from one set of sister ohnologues to another. Indeed, case studies indicate that one set of ohnologues within a cell may be phosphorylated and consequently bind to 14-3-3s when cells are stimulated by insulin for example, while other combinations of sister ohnologues bind to 14-3-3s in response to phosphorylations that are promoted by growth factors, nutrient stress and adrenalin. Partitioning of ohnologues into obligate sub-networks does not seem to be so clear-cut as in *S. cerevisiae* however, as mammalian ohnologues have been identified that are convergence points for regulated interactions with 14-3-3 proteins in response to multiple stimuli ^88, 105– 106^.

These few examples—and the aforementioned ABCDE model of floral development—indicate how the parallelised signalling networks of ohnologues generated via WGDs act as multiple-input multiple-output systems. Collectively, these systems generate different cell phenotypes via differential expression and post-translational switching among sets of ohnologues with different kinetic and regulatory properties.

Deeper understanding of how post-WGD signalling networks operate will underpin advances in understanding polygenic disorders. For instance, mutations in many ohnologue genes are associated with neurological and psychiatric diseases and developmental disorders such as RASopathies ^107, 108^, and there are many examples of heterogeneous patterns of overexpressions and mutations across ohnologue gene families in cancers ^109– 111^. For example, overexpression of insulin receptor substrate 4 (IRS4) drives a subset of breast cancers, while IRS1 and IRS2 are not oncogenic in these cancers, even though all three IRS proteins activate PI 3-kinase–Akt growth signalling. The critical difference is that IRS4 lacks a negative feedback mechanism by which its sisters IRS1 and IRS2 can switch off the pathway via the tyrosine phosphatase SHP2 ^109^. This example illustrates how the parallel signalling pathways generated via WGDs can evolve specific regulatory interconnections, which in principle enable these systems to integrate inputs from multiple sensory stimuli, buffer signal noise via responsive feedback loops, and generate a wider repertoire of phenotypic outcomes than would be possible with the original simple circuit ^112, 113^.

Finally, it should be noted that WGDs do not underpin every evolutionary leap: A WGD was hypothesised to explain why cephalopods (squids, cuttlefish and octopuses) are behaviourally more sophisticated than other molluscs. However, the octopus genome shows no evidence of a WGD. Rather, sensory intelligence in cephalopods is likely to be underpinned by the massively expanded gene families of C2H2 zinc-finger transcription factors and protocadherins that are expressed in their neuronal and sensitive tissues ^114^. It will be fascinating to compare the neural network topologies that have been built from gene families generated via WGDs versus those made of multiple small-scale duplications in humans and octopuses.

## References

[1] Darwin CR: Letter to J.D. Hooker on 22 July 1879, MS DAR.95:485–488. Reference Source

[2] Yeaman S Hodgins KA Lotterhos KE: Convergent local adaptation to climate in distantly related conifers. *Science.* 2016;353(6306):1431–1433. 10.1126/science.aaf7812 27708038

[3] Guan R Zhao Y Zhang H: Draft genome of the living fossil *Ginkgo biloba*. *Gigascience.* 2016;5(1):49. 10.1186/s13742-016-0154-1 27871309 PMC5118899

[4] Šmarda P Veselý P Šmerda J: Polyploidy in a 'living fossil' *Ginkgo biloba*. *New Phytol.* 2016;212(1):11–14. 10.1111/nph.14062 27265838

[5] Scott AD Stenz NW Ingvarsson PK: Whole genome duplication in coast redwood ( *Sequoia sempervirens*) and its implications for explaining the rarity of polyploidy in conifers. *New Phytol.* 2016;211(1):186–193. 10.1111/nph.13930 26996245

[6] Murat F Armero A Pont C: Reconstructing the genome of the most recent common ancestor of flowering plants. *Nat Genet.* 2017;49(4):490–496. 10.1038/ng.3813 28288112

[7] Li Z Baniaga AE Sessa EB: Early genome duplications in conifers and other seed plants. *Sci Adv.* 2015;1(10):e1501084. 10.1126/sciadv.1501084 26702445 PMC4681332

[8] Jiao Y Wickett NJ Ayyampalayam S: Ancestral polyploidy in seed plants and angiosperms. *Nature.* 2011;473(7345):97–100. 10.1038/nature09916 21478875

[9] Clark JW Donoghue PC: Constraining the timing of whole genome duplication in plant evolutionary history. *Proc Biol Sci.* 2017;284(1858): pii: 20170912. 10.1098/rspb.2017.0912 28679730 PMC5524505

[10] Chartier M Löfstrand S von Balthazar M: How (much) do flowers vary? Unbalanced disparity among flower functional modules and a mosaic pattern of morphospace occupation in the order Ericales. *Proc Biol Sci.* 2017;284(1852): pii: 20170066. 10.1098/rspb.2017.0066 28381623 PMC5394665

[11] Amborella Genome Project: The *Amborella*genome and the evolution of flowering plants. *Science.* 2013;342(6165):1241089. 10.1126/science.1241089 24357323

[12] Friis EM Pedersen KR Crane PR: Diversity in obscurity: fossil flowers and the early history of angiosperms. *Philos Trans R Soc Lond B Biol Sci.* 2010;365(1539):369–382. 10.1098/rstb.2009.0227 20047865 PMC2838257

[13] Herendeen PS Friis EM Pedersen KR: Palaeobotanical redux: revisiting the age of the angiosperms. *Nat Plants.* 2017;3:17015. 10.1038/nplants.2017.15 28260783

[14] Ohno S: Gene duplication and the uniqueness of vertebrate genomes circa 1970–1999. *Semin Cell Dev Biol.* 1999;10(5):517–522. 10.1006/scdb.1999.0332 10597635

[15] Wolfe K: Robustness--it's not where you think it is. *Nat Genet.* 2000;25(1):3–4. 10.1038/75560 10802639

[16] Force A Lynch M Pickett FB: Preservation of duplicate genes by complementary, degenerative mutations. *Genetics.* 1999;151(4):1531–1545. 10101175 10.1093/genetics/151.4.1531 PMC1460548

[17] Bowman JL Smyth DR Meyerowitz EM: The ABC model of flower development: then and now. *Development.* 2012;139(22):4095–4098. 10.1242/dev.083972 23093420

[18] Coen ES Meyerowitz EM: The war of the whorls: genetic interactions controlling flower development. *Nature.* 1991;353(6339):31–37. 10.1038/353031a0 1715520

[19] Theißen G Melzer R Rümpler F: MADS-domain transcription factors and the floral quartet model of flower development: linking plant development and evolution. *Development.* 2016;143(18):3259–3271. 10.1242/dev.134080 27624831

[20] Espinosa-Soto C Immink RG Angenent GC: Tetramer formation in *Arabidopsis* MADS domain proteins: analysis of a protein-protein interaction network. *BMC Syst Biol.* 2014;8:9. 10.1186/1752-0509-8-9 24468197 PMC3913338

[21] Silva CS Puranik S Round A: Evolution of the Plant Reproduction Master Regulators LFY and the MADS Transcription Factors: The Role of Protein Structure in the Evolutionary Development of the Flower. *Front Plant Sci.* 2015;6:1193. 10.3389/fpls.2015.01193 26779227 PMC4701952

[22] Wang YQ Melzer R Theissen G: Molecular interactions of orthologues of floral homeotic proteins from the gymnosperm *Gnetum gnemon* provide a clue to the evolutionary origin of ‘floral quartets’. *Plant J.* 2010;64(2):177–190. 10.1111/j.1365-313X.2010.04325.x 21070403

[23] Gramzow L Weilandt L Theissen G: MADS goes genomic in conifers: towards determining the ancestral set of MADS-box genes in seed plants. *Ann Bot.* 2014;114(7):1407–1429. 10.1093/aob/mcu066 24854168 PMC4204780

[24] Chen F Zhang X Liu X: Evolutionary Analysis of MIKC ^c^-Type MADS-Box Genes in Gymnosperms and Angiosperms. *Front Plant Sci.* 2017;8:895. 10.3389/fpls.2017.00895 28611810 PMC5447709

[25] Sauquet H von Balthazar M Magallón S: The ancestral flower of angiosperms and its early diversification. *Nat Commun.* 2017;8: 16047. 10.1038/ncomms16047 28763051 PMC5543309

[26] Chanderbali AS Berger BA Howarth DG: Evolving Ideas on the Origin and Evolution of Flowers: New Perspectives in the Genomic Era. *Genetics.* 2016;202(4):1255–1265. 10.1534/genetics.115.182964 27053123 PMC4905540

[27] Kim YM Kim S Koo N: Genome analysis of *Hibiscus syriacus* provides insights of polyploidization and indeterminate flowering in woody plants. *DNA Res.* 2017;24(1):71–80. 10.1093/dnares/dsw049 28011721 PMC5381346

[28] Mondragón-Palomino M Theissen G: Why are orchid flowers so diverse? Reduction of evolutionary constraints by paralogues of class B floral homeotic genes. *Ann Bot.* 2009;104(3):583–594. 10.1093/aob/mcn258 19141602 PMC2720651

[29] Hane JK Ming Y Kamphuis LG: A comprehensive draft genome sequence for lupin ( *Lupinus angustifolius*), an emerging health food: insights into plant-microbe interactions and legume evolution. *Plant Biotechnol J.* 2017;15(3):318–330. 10.1111/pbi.12615 27557478 PMC5316927

[30] Guo HS Zhang YM Sun XQ: Evolution of the KCS gene family in plants: the history of gene duplication, sub/neofunctionalization and redundancy. *Mol Genet Genomics.* 2016;291(2):739–752. 10.1007/s00438-015-1142-3 26563433

[31] Chansler MT Ferguson CJ Fehlberg SD: The role of polyploidy in shaping morphological diversity in natural populations of *Phlox amabilis*. *Am J Bot.* 2016;103(9):1546–1558. 10.3732/ajb.1600183 27589933

[32] Taylor A Qiu YL: Evolutionary History of Subtilases in Land Plants and Their Involvement in Symbiotic Interactions. *Mol Plant Microbe Interact.* 2017;30(6):489–501. 10.1094/MPMI-10-16-0218-R 28353400

[33] Chanderbali AS Berger BA Howarth DG: Evolution of floral diversity: genomics, genes and *gamma*. *Philos Trans R Soc Lond B Biol Sci.* 2017;372(1713): pii: 20150509. 10.1098/rstb.2015.0509 27994132 PMC5182423

[34] Edger PP Heidel-Fischer HM Bekaert M: The butterfly plant arms-race escalated by gene and genome duplications. *Proc Natl Acad Sci U S A.* 2015;112(27):8362–8366. 10.1073/pnas.1503926112 26100883 PMC4500235

[35] Salman-Minkov A Sabath N Mayrose I: Whole-genome duplication as a key factor in crop domestication. *Nat Plants.* 2016;2; 16115. 10.1038/nplants.2016.115 27479829

[36] Hao M Li A Shi T: The abundance of homoeologue transcripts is disrupted by hybridization and is partially restored by genome doubling in synthetic hexaploid wheat. *BMC Genomics.* 2017;18(1):149. 10.1186/s12864-017-3558-0 28187716 PMC5303294

[37] Otto SP: The evolutionary consequences of polyploidy. *Cell.* 2007;131(3):452–462. 10.1016/j.cell.2007.10.022 17981114

[38] De Storme N Geelen D: Sexual polyploidization in plants--cytological mechanisms and molecular regulation. *New Phytol.* 2013;198(3):670–684. 10.1111/nph.12184 23421646 PMC3744767

[39] Garsmeur O Schnable JC Almeida A: Two evolutionarily distinct classes of paleopolyploidy. *Mol Biol Evol.* 2014;31(2):448–454. 10.1093/molbev/mst230 24296661

[40] Comai L: The advantages and disadvantages of being polyploid. *Nat Rev Genet.* 2005;6(11):836–846. 10.1038/nrg1711 16304599

[41] Mayrose I Zhan SH Rothfels CJ: Methods for studying polyploid diversification and the dead end hypothesis: a reply to Soltis *et al*. (2014). *New Phytol.* 2015;206(1):27–35. 10.1111/nph.13192 25472785

[42] Soltis DE Segovia-Salcedo MC Jordon-Thaden I: Are polyploids really evolutionary dead-ends (again)? A critical reappraisal of Mayrose *et al*. (). *New Phytol.* 2014;202(4):1105–1117. 10.1111/nph.12756 24754325

[43] Mayrose I Zhan SH Rothfels CJ: Recently formed polyploid plants diversify at lower rates. *Science.* 2011;333(6047):1257. 10.1126/science.1207205 21852456

[44] Kitavi M Downing T Lorenzen J: The triploid East African Highland Banana (EAHB) genepool is genetically uniform arising from a single ancestral clone that underwent population expansion by vegetative propagation. *Theor Appl Genet.* 2016;129(3):547–561. 10.1007/s00122-015-2647-1 26743524

[45] Ploetz RC: Fusarium Wilt of Banana. *Phytopathology.* 2015;105(12):1512–1521. 10.1094/PHYTO-04-15-0101-RVW 26057187

[46] Van de Peer Y Mizrachi E Marchal K: The evolutionary significance of polyploidy. *Nat Rev Genet.* 2017;18(7):411–424. 10.1038/nrg.2017.26 28502977

[47] Ma Y Xue H Zhang L: Involvement of Auxin and Brassinosteroid in Dwarfism of Autotetraploid Apple ( *Malus* x *domestica*). *Sci Rep.* 2016;6: 26719. 10.1038/srep26719 27216878 PMC4877651

[48] Tsukaya H: Does ploidy level directly control cell size? Counterevidence from Arabidopsis genetics. *PLoS One.* 2013;8(12):e83729. 10.1371/journal.pone.0083729 24349549 PMC3861520

[49] Ng DW Chen HH Chen ZJ: Heterologous protein-DNA interactions lead to biased allelic expression of circadian clock genes in interspecific hybrids. *Sci Rep.* 2017;7: 45087. 10.1038/srep45087 28345627 PMC5366859

[50] Sahoo T Dzidic N Strecker MN: Comprehensive genetic analysis of pregnancy loss by chromosomal microarrays: outcomes, benefits, and challenges. *Genet Med.* 2017;19(1):83–89. 10.1038/gim.2016.69 27337029

[51] Forstmeier W Ellegren H: Trisomy and triploidy are sources of embryo mortality in the zebra finch. *Proc Biol Sci.* 2010;277(1694):2655–2660. 10.1098/rspb.2010.0394 20444723 PMC2982043

[52] Dong Q Eudeline B Huang C: Commercial-scale sperm cryopreservation of diploid and tetraploid Pacific oysters, *Crassostrea gigas*. *Cryobiology.* 2005;50(1):1–16. 10.1016/j.cryobiol.2004.09.003 15710364

[53] Schwager EE Sharma PP Clarke T: The house spider genome reveals an ancient whole-genome duplication during arachnid evolution. *bioRxi.* 2017 10.1101/106385 PMC5535294 28756775

[54] Kenny NJ Chan KW Nong W: Ancestral whole-genome duplication in the marine chelicerate horseshoe crabs. *Heredity (Edinb).* 2016;116(2):190–199. 10.1038/hdy.2015.89 26419336 PMC4806888

[55] Scannell DR Frank AC Conant GC: Independent sorting-out of thousands of duplicated gene pairs in two yeast species descended from a whole-genome duplication. *Proc Natl Acad Sci U S A.* 2007;104(20):8397–8402. 10.1073/pnas.0608218104 17494770 PMC1895961

[56] Pelin A Selman M Aris-Brosou S: Genome analyses suggest the presence of polyploidy and recent human-driven expansions in eight global populations of the honeybee pathogen *Nosema ceranae*. *Environ Microbiol.* 2015;17(11):4443–4458. 10.1111/1462-2920.12883 25914091

[57] Soltis DE Visger CJ Marchant DB: Polyploidy: Pitfalls and paths to a paradigm. *Am J Bot.* 2016;103(7):1146–1166. 10.3732/ajb.1500501 27234228

[58] Mandaková T Li Z Barker MS: Diverse genome organization following 13 independent mesopolyploid events in Brassicaceae contrasts with convergent patterns of gene retention. *Plant J.* 2017;91(1):3–21. 10.1111/tpj.13553 28370611

[59] Putnam NH Butts T Ferrier DE: The amphioxus genome and the evolution of the chordate karyotype. *Nature.* 2008;453(7198):1064–1071. 10.1038/nature06967 18563158

[60] Session AM Uno Y Kwon T: Genome evolution in the allotetraploid frog *Xenopus laevis*. *Nature.* 2016;538(7625):336–343. 10.1038/nature19840 27762356 PMC5313049

[61] Santini F Harmon LJ Carnevale G: Did genome duplication drive the origin of teleosts? A comparative study of diversification in ray-finned fishes. *BMC Evol Biol.* 2009;9:194. 10.1186/1471-2148-9-194 19664233 PMC2743667

[62] Inoue J Sato Y Sinclair R: Rapid genome reshaping by multiple-gene loss after whole-genome duplication in teleost fish suggested by mathematical modeling. *Proc Natl Acad Sci U S A.* 2015;112(48):14918–14923. 10.1073/pnas.1507669112 26578810 PMC4672829

[63] Suárez-Villota EY Vargas RA Marchant CL: Distribution of repetitive DNAs and the hybrid origin of the red vizcacha rat (Octodontidae). *Genome.* 2012;55(2):105–117. 10.1139/g11-084 22272977

[64] Mendivil Ramos O Ferrier DE: Mechanisms of Gene Duplication and Translocation and Progress towards Understanding Their Relative Contributions to Animal Genome Evolution. *Int J Evol Biol.* 2012;2012: 846421. 10.1155/2012/846421 22919542 PMC3420103

[65] Aury JM Jaillon O Duret L: Global trends of whole-genome duplications revealed by the ciliate *Paramecium tetraurelia*. *Nature.* 2006;444(7116):171–178. 10.1038/nature05230 17086204

[66] Haufler CH: Ever since Klekowski: testing a set of radical hypotheses revives the genetics of ferns and lycophytes. *Am J Bot.* 2014;101(12):2036–2042. 10.3732/ajb.1400317 25480700

[67] Wolfe KH: Origin of the Yeast Whole-Genome Duplication. *PLoS Biol.* 2015;13(8):e1002221. 10.1371/journal.pbio.1002221 26252643 PMC4529243

[68] Marcet-Houben M Gabaldón T: Beyond the Whole-Genome Duplication: Phylogenetic Evidence for an Ancient Interspecies Hybridization in the Baker's Yeast Lineage. *PLoS Biol.* 2015;13(8):e1002220. 10.1371/journal.pbio.1002220 26252497 PMC4529251

[69] Christensen KA Davidson WS: Autopolyploidy genome duplication preserves other ancient genome duplications in Atlantic salmon ( *Salmo salar*). *PLoS One.* 2017;12(2):e0173053. 10.1371/journal.pone.0173053 28241055 PMC5328387

[70] Martin KJ Holland PW: Enigmatic orthology relationships between *Hox* clusters of the African butterfly fish and other teleosts following ancient whole-genome duplication. *Mol Biol Evol.* 2014;31(10):2592–2611. 10.1093/molbev/msu202 24974377 PMC4166920

[71] Furlong RF Holland PW: Were vertebrates octoploid? *Philos Trans R Soc Lond B Biol Sci.* 2002;357(1420):531–544. 10.1098/rstb.2001.1035 12028790 PMC1692965

[72] Schranz ME Mohammadin S Edger PP: Ancient whole genome duplications, novelty and diversification: the WGD Radiation Lag-Time Model. *Curr Opin Plant Biol.* 2012;15(2):147–153. 10.1016/j.pbi.2012.03.011 22480429

[73] Clarke JT Lloyd GT Friedman M: Little evidence for enhanced phenotypic evolution in early teleosts relative to their living fossil sister group. *Proc Natl Acad Sci U S A.* 2016;113(41):11531–11536. 10.1073/pnas.1607237113 27671652 PMC5068283

[74] Byrne KP Wolfe KH: The Yeast Gene Order Browser: combining curated homology and syntenic context reveals gene fate in polyploid species. *Genome Res.* 2005;15(10):1456–1461. 10.1101/gr.3672305 16169922 PMC1240090

[75] Lien S Koop BF Sandve SR: The Atlantic salmon genome provides insights into rediploidization. *Nature.* 2016;533(7602):200–205. 10.1038/nature17164 27088604 PMC8127823

[76] Robertson FM Gundappa MK Grammes F: Lineage-specific rediploidization is a mechanism to explain time-lags between genome duplication and evolutionary diversification. *Genome Biol.* 2017;18(1):111. 10.1186/s13059-017-1241-z 28615063 PMC5470254

[77] Vanneste K Baele G Maere S: Analysis of 41 plant genomes supports a wave of successful genome duplications in association with the Cretaceous-Paleogene boundary. *Genome Res.* 2014;24(8):1334–1347. 10.1101/gr.168997.113 24835588 PMC4120086

[78] Macqueen DJ Johnston IA: A well-constrained estimate for the timing of the salmonid whole genome duplication reveals major decoupling from species diversification. *Proc Biol Sci.* 2014;281(1778):20132881. 10.1098/rspb.2013.2881 24452024 PMC3906940

[79] Bayés À Collins MO Reig-Viader R: Evolution of complexity in the zebrafish synapse proteome. *Nat Commun.* 2017;8: 14613. 10.1038/ncomms14613 28252024 PMC5337974

[80] Furlong RF Graham A: Vertebrate neurogenin evolution: long-term maintenance of redundant duplicates. *Dev Genes Evol.* 2005;215(12):639–644. 10.1007/s00427-005-0023-x 16220265

[81] Locascio A Manzanares M Blanco MJ: Modularity and reshuffling of *Snail* and *Slug* expression during vertebrate evolution. *Proc Natl Acad Sci U S A.* 2002;99(26):16841–16846. 10.1073/pnas.262525399 12482931 PMC139231

[82] Ascencio D Ochoa S Delaye L: Increased rates of protein evolution and asymmetric deceleration after the whole-genome duplication in yeasts. *BMC Evol Biol.* 2017;17(1):40. 10.1186/s12862-017-0895-1 28166720 PMC5294719

[83] Wu P Wang W Duan W: Comprehensive Analysis of the CDPK-SnRK Superfamily Genes in Chinese Cabbage and Its Evolutionary Implications in Plants. *Front Plant Sci.* 2017;8:162. 10.3389/fpls.2017.00162 28239387 PMC5301275

[84] Conant GC Wolfe KH: Turning a hobby into a job: how duplicated genes find new functions. *Nat Rev Genet.* 2008;9(12):938–950. 10.1038/nrg2482 19015656

[85] De Smet R Van de Peer Y: Redundancy and rewiring of genetic networks following genome-wide duplication events. *Curr Opin Plant Biol.* 2012;15(2):168–176. 10.1016/j.pbi.2012.01.003 22305522

[86] Corrochano LM Kuo A Marcet-Houben M: Expansion of Signal Transduction Pathways in Fungi by Extensive Genome Duplication. *Curr Biol.* 2016;26(12):1577–1584. 10.1016/j.cub.2016.04.038 27238284 PMC5089372

[87] Nithianantharajah J Komiyama NH McKechanie A: Synaptic scaffold evolution generated components of vertebrate cognitive complexity. *Nat Neurosci.* 2013;16(1):16–24. 10.1038/nn.3276 23201973 PMC4131247

[88] Tinti M Johnson C Toth R: Evolution of signal multiplexing by 14-3-3-binding 2R-ohnologue protein families in the vertebrates. *Open Biol.* 2012;2(7):120103. 10.1098/rsob.120103 22870394 PMC3411107

[89] Huminiecki L Heldin CH: 2R and remodeling of vertebrate signal transduction engine. *BMC Biol.* 2010;8:146. 10.1186/1741-7007-8-146 21144020 PMC3238295

[90] Manning G Whyte DB Martinez R: The protein kinase complement of the human genome. *Science.* 2002;298(5600):1912–1934. 10.1126/science.1075762 12471243

[91] Ruiz i Altaba A Mas C Stecca B: The Gli code: an information nexus regulating cell fate, stemness and cancer. *Trends Cell Biol.* 2007;17(9):438–447. 10.1016/j.tcb.2007.06.007 17845852 PMC2601665

[92] Makino T McLysaght A: Ohnologs in the human genome are dosage balanced and frequently associated with disease. *Proc Natl Acad Sci U S A.* 2010;107(20):9270–9274. 10.1073/pnas.0914697107 20439718 PMC2889102

[93] Pérez-Bercoff A Makino T McLysaght A: Duplicability of self-interacting human genes. *BMC Evol Biol.* 2010;10:160. 10.1186/1471-2148-10-160 20509897 PMC2894830

[94] Wu X Qi X: Genes encoding hub and bottleneck enzymes of the *Arabidopsis* metabolic network preferentially retain homeologs through whole genome duplication. *BMC Evol Biol.* 2010;10:145. 10.1186/1471-2148-10-145 20478072 PMC2880986

[95] Pires JC Conant GC: Robust Yet Fragile: Expression Noise, Protein Misfolding, and Gene Dosage in the Evolution of Genomes. *Annu Rev Genet.* 2016;50:113–131. 10.1146/annurev-genet-120215-035400 27617972

[96] Nguyen Ba AN Strome B Hua JJ: Detecting functional divergence after gene duplication through evolutionary changes in posttranslational regulatory sequences. *PLoS Comput Biol.* 2014;10(12):e1003977. 10.1371/journal.pcbi.1003977 25474245 PMC4256066

[97] Wang Z Ding G Geistlinger L: Evolution of protein phosphorylation for distinct functional modules in vertebrate genomes. *Mol Biol Evol.* 2011;28(3):1131–1140. 10.1093/molbev/msq268 20956806

[98] Ruiz i Altaba A: Hedgehog signaling and the Gli code in stem cells, cancer, and metastases. *Sci Signal.* 2011;4(200):pt9. 10.1126/scisignal.2002540 22114144

[99] Grant SG: The molecular evolution of the vertebrate behavioural repertoire. *Philos Trans R Soc Lond B Biol Sci.* 2016;371(1685):20150051. 10.1098/rstb.2015.0051 26598730 PMC4685586

[100] Conant GC Wolfe KH: Functional partitioning of yeast co-expression networks after genome duplication. *PLoS Biol.* 2006;4(4):e109. 10.1371/journal.pbio.0040109 16555924 PMC1420641

[101] Palma M Dias PJ Roque FC: The *Zygosaccharomyces bailii* transcription factor Haa1 is required for acetic acid and copper stress responses suggesting subfunctionalization of the ancestral bifunctional protein Haa1/Cup2. *BMC Genomics.* 2017;18(1):75. 10.1186/s12864-016-3443-2 28086780 PMC5234253

[102] Mattenberger F Sabater-Muñoz B Toft C: The Phenotypic Plasticity of Duplicated Genes in *Saccharomyces cerevisiae* and the Origin of Adaptations. *G3 (Bethesda).* 2017;7(1):63–75. 10.1534/g3.116.035329 27799339 PMC5217124

[103] Williams KM Liu P Fay JC: Evolution of ecological dominance of yeast species in high-sugar environments. *Evolution.* 2015;69(8):2079–2093. 10.1111/evo.12707 26087012 PMC4751874

[104] Conant GC Wolfe KH: Increased glycolytic flux as an outcome of whole-genome duplication in yeast. *Mol Syst Biol.* 2007;3:129. 10.1038/msb4100170 17667951 PMC1943425

[105] Chen S Murphy J Toth R: Complementary regulation of TBC1D1 and AS160 by growth factors, insulin and AMPK activators. *Biochem J.* 2008;409(2):449–459. 10.1042/BJ20071114 17995453

[106] Johnson C Crowther S Stafford MJ: Bioinformatic and experimental survey of 14-3-3-binding sites. *Biochem J.* 2010;427(1):69–78. 10.1042/BJ20091834 20141511 PMC2860806

[107] Bayés A van de Lagemaat LN Collins MO: Characterization of the proteome, diseases and evolution of the human postsynaptic density. *Nat Neurosci.* 2011;14(1):19–21. 10.1038/nn.2719 21170055 PMC3040565

[108] Aoki Y Niihori T Inoue S: Recent advances in RASopathies. *J Hum Genet.* 2016;61(1):33–39. 10.1038/jhg.2015.114 26446362

[109] Ikink GJ Boer M Bakker ER: IRS4 induces mammary tumorigenesis and confers resistance to HER2-targeted therapy through constitutive PI3K/AKT-pathway hyperactivation. *Nat Commun.* 2016;7: 13567. 10.1038/ncomms13567 27876799 PMC5122961

[110] Tinti M Dissanayake K Synowsky S: Identification of 2R-ohnologue gene families displaying the same mutation-load skew in multiple cancers. *Open Biol.* 2014;4(5):140029. 10.1098/rsob.140029 24806839 PMC4042849

[111] Newlaczyl AU Coulson JM Prior IA: Quantification of spatiotemporal patterns of Ras isoform expression during development. *Sci Rep.* 2017;7: 41297. 10.1038/srep41297 28117393 PMC5259795

[112] Kafri R Levy M Pilpel Y: The regulatory utilization of genetic redundancy through responsive backup circuits. *Proc Natl Acad Sci U S A.* 2006;103(31):11653–11658. 10.1073/pnas.0604883103 16861297 PMC1513536

[113] Baker CR Hanson-Smith V Johnson AD: Following gene duplication, paralog interference constrains transcriptional circuit evolution. *Science.* 2013;342(6154):104–108. 10.1126/science.1240810 24092741 PMC3911913

[114] Albertin CB Simakov O Mitros T: The octopus genome and the evolution of cephalopod neural and morphological novelties. *Nature.* 2015;524(7564):220–224. 10.1038/nature14668 26268193 PMC4795812

